# Burnout Syndrome Among Spanish Professionals Dedicated to Implant Dentistry: An Observational Study

**DOI:** 10.3390/healthcare13141724

**Published:** 2025-07-17

**Authors:** Ángel-Orión Salgado-Peralvo, Andrea Uribarri, Eugenio Velasco-Ortega, José López-López, Álvaro Jiménez-Guerra, Loreto Monsalve-Guil, Jesús Moreno-Muñoz, José-Luis Rondón-Romero, Iván Ortiz-García, Enrique Núñez-Márquez

**Affiliations:** 1 Department of Surgery and Medical-Surgical Specialties, Faculty of Dentistry and Medicine, University of Santiago de Compostela, 15782 Santiago de Compostela, Spain; 2 Independent Researcher, D02 PE00 Dublin, Ireland; auribarride@gmail.com; 3 Department of Stomatology, Faculty of Dentistry, University of Seville, 41009 Seville, Spain; evelasco@us.es (E.V.-O.); alopajanosas@hotmail.com (Á.J.-G.); lomonsalve@hotmail.es (L.M.-G.); jolurr001@hotmail.com (J.-L.R.-R.); ivanortizgarcia1000@hotmail.com (I.O.-G.); enrique_aracena@hotmail.com (E.N.-M.); 4 Department of Odontostomatology, Faculty of Dentistry, University of Barcelona, 08907 Barcelona, Spain; 18575jll@gmail.com

**Keywords:** burnout, psychological, burnout, professional, caregiver burden, Maslach burnout inventory, dental implants, surveys and questionnaires

## Abstract

**Background**: Burnout syndrome (BS) is an occupational condition resulting from chronic stress, characterized by three dimensions, emotional exhaustion (EE), depersonalization (DE), and diminished personal accomplishment (PA), particularly prevalent in caregiving professions such as healthcare. The aim of this study is to analyse the prevalence of BS among Spanish dental implantology specialists, along with the impact of demographic, educational, and professional aspects. **Methods**: This is a cross-sectional observational study based on the STROBE (Strengthening the Reporting of Observational Studies in Epidemiology) guidelines and was open to respondents from May to December 2024. An electronic survey based on the Maslach Burnout Inventory—Human Services Survey (MBI–HSS) was sent to members of the Spanish Society of Implants. The data were analysed using descriptive analysis. **Results**: A total of 305 participants (20.9%) (31.5% females and 68.5% males) completed the questionnaire. The prevalence of BS was 4.3%; however, 61.0% of the dentists showed signs of suffering from the syndrome. The mean values of EE were “average” (20.3 ± 13.8) and of DE and EE “low” (5.1 ± 5.9, and 32.5 ± 14.5, respectively). The factors significantly associated with suffering from BS were being female and having more than 20 years of experience in dental implant treatments. **Conclusions**: It is advisable to conduct instructive and awareness-raising initiatives among dental professionals to promote an awareness of their mental health, ultimately aiming at preserving their physical and emotional well-being while delivering optimal care to their patients.

## 1. Introduction

The concept of burnout was first articulated by Freudenberger in 1974 [1] to describe the physical, psychological, and social symptoms arising from energy demands that surpass a worker’s resources, particularly noted in individuals with high, often unattainable, self-expectations. Maslach and Leiter [2] identified that burnout syndrome (BS) consists of three dimensions, emotional exhaustion (EE), depersonalization (DE), and a reduction in personal accomplishment (PA), particularly prevalent in caregiving professions like healthcare, where a substantial portion of the professional–patient interaction centres on the patient’s issues, often accompanied by emotions such as anger, shame, fear, or despair.

The ongoing exposure to the previously mentioned conditions induces persistent stress, resulting in “distancing” and negative thoughts, which subsequently diminish professional performance [3]. The primary symptoms of high EE include fatigue—both physical and mental—following the workday, a lack of desire to attend work, difficulty concentrating, insomnia, frequent headaches, gastrointestinal disorders, and resistance to change. High DE is characterized by a negative, indifferent, and/or cynical attitude toward patients or other colleagues in the profession, which results in an emotional disconnection and insensitivity to the work they perform [4]; and of low PA, a feeling of dissatisfaction, failure, anger, and resentment, even a feeling of guilt and lack of self-respect, as well as discouragement and indifference [5]. Nonetheless, it is not solely an individual issue; it can also be transmitted to the medical staff and the patient, and vice versa [6], adversely affecting the dental practices, elevating absenteeism, clinical errors, and poor patient service [5].

Burnout is included in the 11th Revision of the International Classification of Diseases (ICD-11) as an occupational phenomenon, and it is defined as, “a syndrome conceptualized as resulting from chronic workplace stress that has not been successfully managed”. It is not classified as a medical condition, as it is described in the chapter, “Factors influencing health status or contact with health services”, which includes reasons for which people contact health services, but that are not classed as illnesses or health conditions [7].

Furthermore, certain authors may regard BS as a stage in the development of depression [8]. Given that their symptoms correspond to the conventional spectrum of conditions linked to depression and anxiety, they can typically manifest as cardiovascular diseases in men and musculoskeletal disorders in women [9].

Numerous studies indicate that 10% to 15% of dentists are at significant risk of experiencing BS [10,11,12,13,14,15]. Idiosyncratic characteristics associated with this profession encompass time constraints, challenging interactions with patients, consultation management, financial issues, diminished work–life balance, and a lack of professional perspective. In certain instances, this illness may further present with harmful behaviours, such as substance abuse or an elevated risk of suicide [16].

The main aim of this study is to assess the incidence of BS among Spanish implant dentistry professionals and examine the influence of various demographic, academic, and professional factors on it.

## 2. Materials and Methods

### 2.1. Study Design

A cross-sectional observational study was carried out following STROBE (Strengthening the Reporting of Observational Studies in Epidemiology) guidelines [17]. The study was performed in compliance with relevant laws and institutional guidelines and has been approved by the Ethics Committee of the CEIm San Carlos Clinic Hospital (Madrid, Spain) (protocol code: 24/280-E; 12 April 2024).

### 2.2. Questionnaire

The survey was sent to all members of the Spanish Society of Implants (SEI), who did not express their wish against receiving e-mails (n = 1460), through Google Drive, and was open to respondents from May to December 2024, during which time 4 reminders were sent so that those who had not answered the questionnaire could do so. Completion of the survey implied the participant’s consent to the gathering of this information. The final sample size comprised the experts who decided to completely reply to the survey. Each respondent could only answer one electronic survey once. Participants were not provided with any specific incentive to complete this survey.

No selection bias was expected, given the electronic survey was sent to all SEI members. A validated survey was utilized, therefore eliminating any information bias. The form consisted of two sections of compulsory questions (Table S1), preventing progression to the subsequent question without answering the preceding one. The first block consisted of 11 general questions related to the surveyed professionals, such as demographic, academic, and professional data.

The second block represents the Maslach Burnout Inventory—Human Services Survey [3] (MBI–HSS), a validated questionnaire assessing three dimensions: firstly, 9 questions regarding the respondent’s (1) EE, measuring the experience of emotional fatigue due to work demands (maximum score [MS] = 54 points). This MS is calculated by multiplying the number of questions (9) by the highest possible score per question (6); secondly, 5 questions evaluating (2) DE—the extent to which individuals exhibit attitudes of coldness and detachment (MS = 30 points); and thirdly, 8 questions analysing (3) PA—feelings of self-confidence and personal fulfilment at work (MS = 48 points).

Each item asks respondents to describe their feelings on a 7-point Likert-type scale, ranging from (0) “never having those feelings” to (6) “having those feelings every day. Based on the score assigned to each dimension of the MBI–HSS, the degree of BS experienced by the respondent is determined. High scores in the first two dimensions (EE ≥ 27; DE ≥ 10) and low scores in the third (PA ≤ 33) define the BS. Likewise, the degree of impairment would decrease or increase in the case of presenting signs of BS in one or two dimensions, respectively (Table 1).

Psychometric properties of the MBI–HSS were checked in dentists by Bassam et al. [18] (2023) through Cronbach’s alphas, with the subsequent values: EE = 0.855; DE = 0.823; and PA = 0.667. The results of the test–retest reliability assessment demonstrated the strong reproducibility of the MBI–HSS [EE, ICC = 0.927 (0.845–0.966), *p*-value < 0.0001; PA, ICC = 0.963 (0.921–0.983), *p*-value < 0.001; DP, ICC = 0.764 (0.497–0.889), *p*-value < 0.0001]. The exploratory factor analysis of the MBI–HSS yielded three psychometrically robust sub-domains representing dimensions of EE, DE, and PA, which explained 57.8% of the scale’s total variance.

### 2.3. Clinical Relevance

BS adversely affects the mental and physical well-being of dentists and maxillofacial surgeons and their medical teams, as well as the quality of care delivered to patients. Consequently, it is essential to ascertain the prevalence of this syndrome among experts in implant dentistry to comprehend its extent and the potential to enhance their quality of life and the dental treatments that they provide.

### 2.4. Sample Size Calculation

The population is constituted by the total number of SEI members (n = 1460). With a power of 90%, a confidence level of 95%, and a margin of error of 5%, a total of 305 respondents were deemed necessary for the analysis to detect significant differences.

### 2.5. Statistical Analysis

The collected data were analysed using IBM^®^ SPSS Statistics v.26 software (IBM^®^ Corp., Armonk, NY, USA). Descriptive statistics were utilized to report the general results of the study. For the cross-tabulations concerning quantitative variables, a normality test was conducted, observing that none of the variables followed a normal distribution, thus applying non-parametric tests. The Mann–Whitney U test was used for the cross-tabulations regarding the dichotomous variables, and the Kruskal–Wallis test for the variables with more than two categories. In cases where the latter was significant, the Mann–Whitney U test was applied in pairwise comparisons to see which groups made a difference. Regarding the intersections between qualitative variables, the Chi-squared test was conducted. To determine the groups that made a difference, Haberman’s standardised residuals were used, obtaining the significance of the cells independently. The variables from block I were taken as independent, and EE, DE, and PA as dependent. A *p*-value < 0.05 was considered statistically significant.

## 3. Results

The survey was responded by a total number of 305 participants; thus, the response rate was 20.9%, which was considered an appropriate number.

### 3.1. Descriptive Data

The survey was answered by 209 men (68.5%) and 96 women (31.5%). The predominant age of the participants was 31 to 40 years (28.5%) and 41 to 50 years (25.2%). Most of the surveyed professionals were dentists (81.0%) and, to a lesser extent, stomatologists (17.0%) and maxillofacial surgeons (2.0%). Most of them had studied a master’s degree related to implant dentistry (57.0%) and had experience more than 20 years (36.4%) or 5 to 15 years (27.9%) in this type of treatments. The professionals that participated had placed more than 101 dental implants per year (61.3%) and did not practice exclusively in dental implant treatments (85.9%). A total of 88.5% of respondents are employed in urban settings, predominantly at their own practices (43.9%), while 64.3% fulfil their positions across many workplaces. Most of the surveyed dental professionals work between 33 to 40 h per week (36.7%) or exceed 40 h per week (37.0%) (Table 2).

### 3.2. Main Results

**Figure 1:**
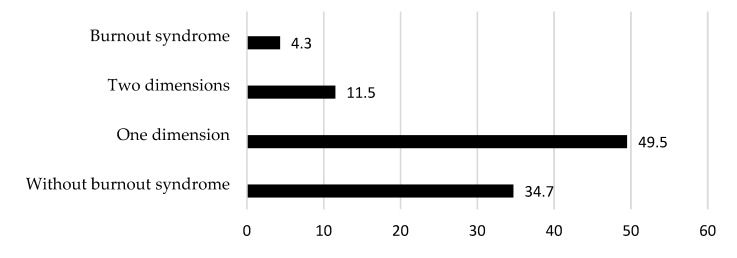
Distribution of participants with BS, with some signs (i.e., high values of EE, DE, or low values of PA), or without BS.

The prevalence of professionals dedicated to implant dentistry who exhibited BS was 4.3% (n = 13/305), significantly affecting more women than men (8.3% vs. 2.4%, respectively; *p* < 0.05). However, 61.0% of the professionals showed signs of suffering from the syndrome (one dimension: 49.5%; two dimensions: 11.5%). Therefore, 34.7% (n = 106) showed no signs of suffering from BS (Figure 1).

**Figure 2:**
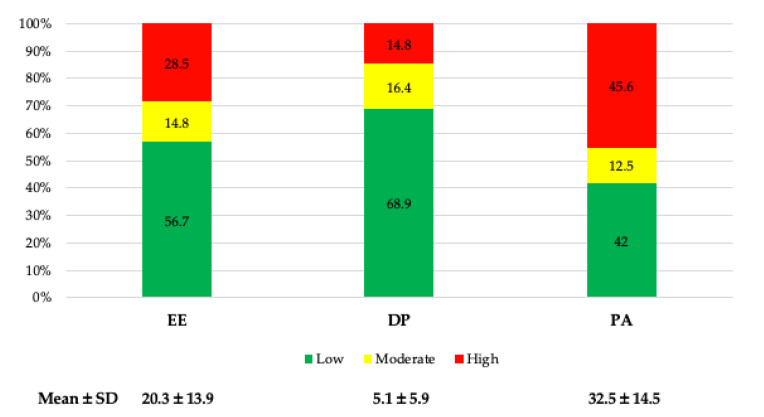
Dimensions of BS (EE, emotional exhaustion; DE, depersonalization; PA, personal accomplishment; SD, standard deviation).

A total of 28.5% exhibited high levels of EE and 14.8% of DE, and 42% exhibited low PA. Despite this, what prevailed the most were low levels of EE and DE (56.7% and 68.9%, respectively) and high levels of PA (45.6%) (Figure 2).

Overall, the average values of EE were within the “average” range (20.3 ± 13.9), while those of DE and PA were “low” (5.1 ± 5.9, and 32.5 ± 14.5, respectively) (Table 3).

Individuals aged over 60 and professionals with over 20 years of experience exhibited markedly lower EE and DE values relative to other age cohorts (*p* < 0.05), with no discernible variations in PA. The quality of undergraduate education and graduate training did not exert a substantial influence. Additional factors that did not affect these parameters included exclusiveness in implant dentistry procedures, the job environment (rural or urban), and the weekly working hours. Respondents working at their own practice or those not employed at numerous centres exhibited significantly elevated PA levels (*p* < 0.05). Table 4 presents the mean values for the EE, DE, and PA variables.

## 4. Discussion

Studies have confirmed that dentists were significantly more likely to experience BS symptoms than any other medical profession group [19,20]. The dental profession has been associated with stress on account of a variety of factors, including the isolated character of the dentist in their workplace and the issues that arise from the functioning of their work team, pressures to meet professional and work-related goals [21], an extended work schedule [21,22]; as well as operational factors, such as exceptional precision while the eyes concentrate on distinct points and the delicate movements of the fingers [23], a noisy medical environment, difficult work postures, prolonged surgical procedures, a narrow operating space, and a desire for technical perfection [24]. Conversely, the patient can also be a source of stress, as some patients exhibit anxiety and/or confrontational behaviour, the time allotted to each patient is limited, and it is necessary to establish a relationship of trust and loyalty with the patient, as well as to ensure the patient is satisfied with the treatment, since they can freely choose between dentists [21]. It is crucial to note that patients expect dental services to be accurate and predictable, which may lead to a lack of appreciation for the dental professional’s efforts. Typically, dissatisfaction is expressed before gratitude for a treatment that is executed effectively. This context is framed within a reality of constantly changing new technologies, as well as work methods [23] that demand the continuous upgrading of dental professionals. However, it is also essential to note that patients have been more likely to file legal claims following oral implant treatments in recent decades. In this regard, a study conducted in Spain examined the demand for dental treatments from 1993 to 2007, revealing that 43.8% were attributable to oral surgery procedures, with 55.6% of them related to dental implant treatments. In 71.4% of the cases, the cause of penalty was directed against the implant dentist, with economic compensations varying from EUR 6000 to EUR 240,000 [25].

These factors provide an ideal environment for the development of chronic occupational stress, which exacerbates the likelihood of these professionals experiencing mental health disorders, including anxiety, depression, and exhaustion, thereby diminishing their quality of life [22]. Due to the correlation between BS and patient safety incidents, it was imperative to examine BS among oral implantologists to intervene before the safety of patients was compromised [26], since implant dentistry malpractice can result in more deleterious outcomes for patients than those associated with other dental specialties [25].

**Figure 3:**
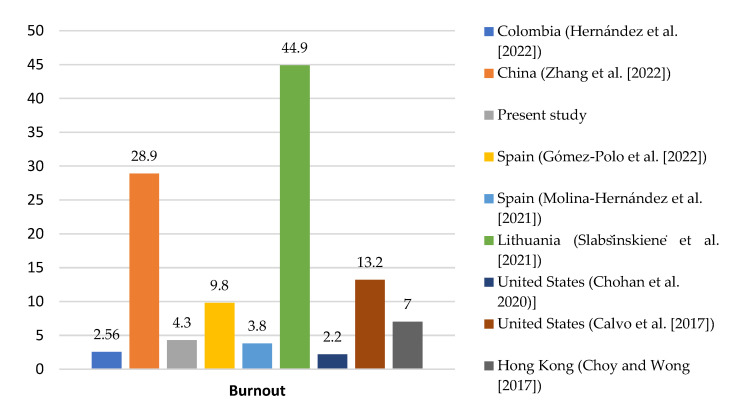
Comparison of the prevalence of BS among different countries in the last 10 years (February 2015 to February 2025) [4,5,8,21,22,28,29].

In the present research, a BS rate of 4.3% has been described, lower than the average of 14.05% reported by studies published in the last 10 years [5,8,21,27,28,29] (Figure 3).

**Figure 4:**
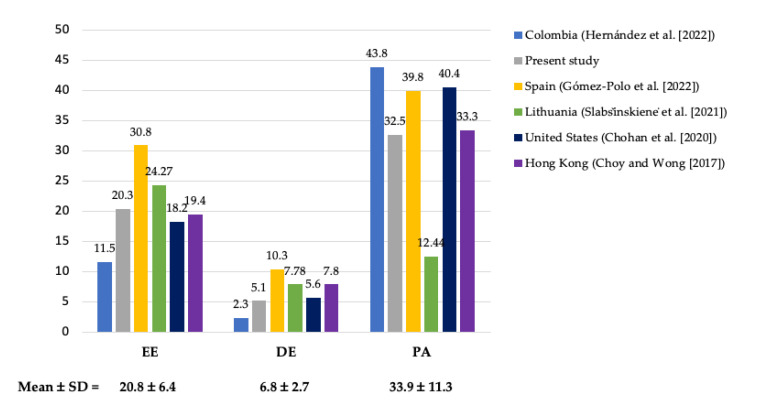
Comparison of BS dimensions among different countries over the past 10 years (February 2015 to February 2025) (EE, emotional exhaustion; DE, depersonalization; PA, personal accomplishment) [4,5,8,21,28].

Nevertheless, a significant number of professionals (61%) exhibited symptoms of BS, indicating a high risk of developing the syndrome. This figure is higher than that reported in a study conducted in the USA (36.3%) [8]. In terms of BS dimensions, values close to the average of other studies were obtained, that is, an EE value of 20.3 ± 13.8 compared to the average of 20.8 ± 6.4 (both in the “average” range), a DE of 5.1 (“low”) vs. 6.8 ± 2.7 (“average”), and a PA of 32.5 vs. 33.9 ± 11.3 (both in the “low” range) (Figure 4).

It is critical to mention that Zhang et al. [29] included the mean of the values for each dimension of BS, so the sum of each dimension could not be obtained for comparison. Molina-Hernández et al. [27] did not provide the values for each of the dimensions.

A relevant fact is that this is the only research conducted entirely on implantologists, while the rest of the studies included a variable number of these dental professionals. More specifically, a study conducted in China included 4.0% of implantologists, in Spain 26.2% of surgeons and periodontists, and in Colombia, 18%. Some authors did not specify these data [28].

The results of this study indicate that the prevalence of BS was significantly higher among professionals who completed a master’s degree in oral implantology (57%), with a rate of 4.6% (*p* < 0.05). This contrasts with the data from Hong Kong, where professionals without postgraduate qualifications have a fivefold increased risk of BS (Odds Ratio [OR] = 5.08; 95% CI: 1.09–23.61; *p* = 0.038) [4]. Professionals could be better prepared for the growing expectations and challenges of patients and society by acquiring a more advanced postgraduate education, which would update their knowledge and skills. Despite this, dentists who have completed advanced training might be subject to increased job expectations, such as reaching a higher income or a higher status, which can have a detrimental impact on their mental health if these expectations are not met [30,31]. More specifically, in Spain, the competitiveness among professionals is very high, with a ratio three times higher than what is recommended by the World Health Organisation (2.94 dentists vs. 1 dentist for every 3500 inhabitants, respectively) [32]. However, future data are not as promising, with the average number of dental graduates per 100,000 residents at 3.62 (8th position in the ranking), which is higher than the European average of 3.20, according to recent Eurostat data [33]. This situation suggests the possibility that Spanish dentists may be overqualified without any significant improvement in their working conditions, which could lead to frustration and anxiety.

The low prevalence of EE in this study may be attributed to the fact that professionals with this syndrome are less likely to respond to 21st-century surveys [34] or that the sample consisted of approximately one-quarter (24.3%) of professionals over the age of 60. In this regard, it was observed that individuals over the age of 60 (which is associated with a treatment experience of over 20 years in these treatments) had significantly lower EE and DE values than the other age groups (*p* < 0.05). However, there were no differences in PA values, even though they had higher values. These results are consistent with those described by other authors [4,21,22]. This may be because of the number of years worked significantly improves decision-making and job satisfaction, which can lower the personal perception of stress [27]. Additionally, they may show a more solid financial position and more stable employment conditions [21]. In contrast, increased BS among young individuals has been associated with shorter work experience [35], while individuals in their mid-career are at the greatest risk for BS due to extended working hours, minimal work–life balance, and elevated rates of EE [36]. These data are in line with those found in our research, with the highest EE values described in professionals aged 31 to 40 years (24.11 ± 14.33), followed by those aged 41 to 50 years (21.55 ± 14.04), and with those obtained in the USA where it was observed that professionals with 11 to 20 years of experience have 2.99 times more DE than those with ≤10 years (*p* = 0.026) [8].

The profile of a professional who exhibits BS or, alternatively, high indications of suffering from it, is a woman between the ages of 31 and 40 who has completed a master’s degree in oral implantology, has a degree in dentistry, has limited experience in these treatments (up to 5 years), places up to 50 implants annually, works in a rural area, and works more than 40 h per week. In this regard, it was observed that women were significantly more affected by BS than men (8.3% vs. 2.4%, *p* < 0.05). In other studies conducted in Spain, women reported a higher level of tension and a lower perception of the work environment than men [27], while another study only observed higher EE values in women (OR = 1.38; *p* = 0.05) [5]. This may be attributable to the fact that men demonstrate substantially higher values in terms of vigour (describing “high levels of energy and resilience”) and absorption (i.e., “being totally and happily immersed in one’s work”) analysed using the Utrecht Work Engagement Scale (UWES) compared to women [22]. In contrast, other studies did not observe differences in the subcomponents of BS [8,21,22,23] or they did not provide this data [4]. To properly assess the possible gender differences, it would be advisable to have more information regarding family situation, pregnancy, and work–life balance, among others.

It has also been observed that the risk of experiencing BS is significantly associated with work engagement (Pearson χ^2^ test = 22.51; *p* < 0.0001), such that 39.3% of professionals with low engagement presented a high BS risk, while none with high work engagement did [22]. In this context, the incidence of EE is substantially reduced by the sense of vocation that one experiences for their chosen profession [23]. This can lead to an enhancement in the work environment, which in turn decreases the incidence of BS among professionals (Pearson correlation coefficient [r] = −0.18; *p* < 0.001) [27]. This is evidenced by the fact that owning one’s own dental practice and exclusively working within it was linked to significantly lower levels of EE and DE (18.17 ± 13.33 and 4.05 ± 5.18) than working for others (22.38 ± 14.05; *p* < 0.05, and 6.07 ± 6.83; *p* < 0.05, respectively) or having one’s own dental practice while simultaneously working for others (21.62 ± 13.92; *p* < 0.05, and 5.73 ± 5.78; *p* < 0.01, respectively). The same observation was made by Gómez-Polo et al. [5] in their study conducted in Spain, where a substantially higher proportion of dentists with signs of BS were observed among those who do not own a dental practice compared to those who do (*p* = 0.008 and *p* < 0.001, respectively). Conversely, it appears that the likelihood of experiencing elevated EE is reduced by 38% when employed in a private dental practice with multiple dentists, as opposed to when employed as the sole dentist (*p* = 0.037) [8]. This can result in the professional feeling less isolated and, in a sense, the collaboration with other colleagues acting as a release.

The key emerging factors of this syndrome are chronic interpersonal stressors at the job [37]. In this regard, a study conducted on dentists in China revealed a prevalence of 25.7% of psychological distress, with the multivariable analysis showing a significant increase in risk due to lower income, BS (OR = 2.171; *p* < 0.001), high job stress (OR = 3.017; *p* < 0.001), career-choice regret (OR = 2.05; *p* < 0.001), and lack of sufficient personal time (OR = 1.735; *p* = 0.004) [29]. Similar data were reported in a study conducted in Hong Kong. Their EE values significantly increased as scores related to time-related stressors (*p* < 0.001) and job-related stressors (*p* < 0.001) increase; DP was associated with job-related stressors (*p* < 0.001) and with private dentists working in residential areas compared to those working in commercial areas (*p* = 0.005). The only factor associated with lower PA was high values in income-related stressors (*p* < 0.001). These authors observed that professionals who score high on job-related stressors had almost 4 times the risk of experiencing BS (OR = 3.74; 95% CI: 1.77–7.87; *p* = 0.001) [4].

The current study revealed no substantial impact of weekly working hours; nonetheless, professionals working over 40 h per week exhibited a markedly higher average EE of 23.84 ± 14.72 compared to those working 33 to 40 h (19.22 ± 12.90; *p* < 0.05) and 25 to 32 h (16.78 ± 11.76; *p* < 0.01). This finding aligns with earlier research indicating that professionals working over 40 h per week have a 10.59-fold increased chance of experiencing high EE compared to those working fewer than 20 h (*p* = 0.023) [8]. Another factor associated with a significantly elevated EE was the placement of fewer than 50 implants annually (26.23 ± 15.51), in contrast to other categories (51 to 100 implants/year: 18.98 ± 13.20; *p* < 0.05; 101 to 200 implants/year: 19.30 ± 13.00; *p* < 0.01; >200 implants/year: 18.99 ± 13.23; *p* < 0.01), potentially attributable to dissatisfaction with the expectations associated with a commitment to implantology. This is directly related to economic compensation, since increased dissatisfaction in this area is highly correlated with higher EE (*p* = 0.0483) and the probability of suffering BS [29].

A longitudinal study conducted on Dutch dentists revealed that EE is an early sign of BS that precedes DE and low levels of PE [38]; therefore, it is recommended to periodically complete the MBI–HSS to identify early signs of suffering from BS and, consequently, establish preventive measures. In this regard, it is advisable to work 25 to 32 h per week, as they exhibited significantly lower EE values than those who worked over 40 h per week (16.78 ± 11.76 vs. 23.84 ± 14.72, respectively; *p* < 0.01). Additionally, the levels of DE (3.94 ± 4.15) were reduced, while PA (31.50 ± 14.20) were increased. Another protective factor is the presence of one’s own clinic, which results in a substantial decrease in EE and DE levels and the highest PA scores when contrasted with working for another individual, or working for another individual and having one’s own clinic. This may be attributable to the perception of possessing a greater “control of one’s own working situation” [4], and to have the autonomy to decide on the number of working hours, personnel, and supplies, among other factors [27].

### 4.1. Limitations

This study had several limitations. Firstly, due to the nature of the survey, the authenticity of the data supplied by respondents cannot be ascertained, as certain personal enquiries may have been answered subjectively, even if the survey was done anonymously. Secondly, responses to anxiety and stress in the workplace are not linear; they may exacerbate or attenuate at various times, influencing the documented prevalence rate. Thirdly, numerous significant factors associated with psychological distress may not have been captured in this survey. Fourthly, although the response rate obtained (20.9%) may be considered low, it is comparable to that obtained in similar surveys included in the present study (range 9.3% [27] to 71.7% [28]). This lower participation can be attributed to the usual care burden of these professionals and the sensitive nature of some questions in the questionnaire. Nevertheless, the obtained sample adequately reflects the distribution by age, sex, and specialty within the target population, suggesting a reasonable representativeness. Even so, a possible non-response bias cannot be ruled out, and therefore, the results should be interpreted with caution. Future studies with higher participation rates will allow for the confirmation of these findings. Lastly, a substantial proportion of professionals experiencing BS may have been unwilling to participate in the study, potentially leading to an underestimation of the BS prevalence reported in this research. In this regard, it has been suggested that depressed people may be less likely to respond to surveys than non-depressed people [34].

### 4.2. Implications of the Study

The results of the present study are highly relevant, as 61.0% of the professionals exhibited symptoms of the syndrome, despite the low prevalence of BS (4.3%). This implies that, in the absence of suitable strategies for its diagnosis and treatment, these professionals may experience a decline in their mental and physical health, which would have negative repercussions for them and their patients. Additionally, the potential for a decline in their dental and stomatological care would be detrimental. Periodic completion of the MBI–HSS is advised to prevent this. In this manner, it is possible to identify changes in the dimensions of BS (EE, DE, and/or PA) at an early stage and implement preventive measures to restore these levels. It is advised that stress management strategies be taught in university teaching programs and by professional dental associations, with the intention of encouraging the request for professional assistance when tension exceeds one’s capacity to cope. Conversely, it is prudent to allocate time for oneself and cultivate a healthy lifestyle, which fosters both physical and mental well-being. Finally, it is imperative to implement organizational and structural measures to enhance the work environment, thereby achieving more effective and long-term changes.

### 4.3. Recommendations for Further Research

Further research should be conducted with a longitudinal approach in order to repeat the survey at different time periods with the same sample of professionals, thereby being able to determine the average prevalence values of BS, as well as the levels of EE, DE, and PA, enabling the acquisition of values closer to reality and understanding the effect of implementing alleviating and/or preventive measures.

## 5. Conclusions

The prevalence of BS among Spanish professionals dedicated to implant dentistry is 4.3%, although only 34.7% showed no signs of suffering from it. The mean values of EE were found within the average, while those of DE and PA were in the low range. The factors significantly associated with suffering from BS were being female and having more than 20 years of experience in implant treatments. Based on the collected data, it is recommended to carry out informative and awareness-raising activities among dental implant dentists to foster a concern for their mental health, with the goal being their physical and emotional well-being, as well as ensuring the best possible care for their patients.

## Figures and Tables

**Table 1:** Reference values of the dimensions evaluated in the MBI–HSS.

Dimension	Low	Average	High	Signs of BS ^4^
EE ^1^	0–18	19–26	27–54	≥27
DE ^2^	0–5	6–9	10–30	≥10
PA ^3^	0–33	34–39	40–48	≤33

^1^ Emotional exhaustion; ^2^ depersonalization; ^3^ personal accomplishment; ^4^ burnout syndrome.

**Table 2:** General data of the surveyed dental specialists.

Variable	Specification	N ^1^	% ^2^
Gender	Female	96	31.5
	Male	209	68.5
Age (years)	≤30	28	9.2
	31–40	87	28.5
	41–50	77	25.2
	51–60	39	12.8
	>60	74	24.3
University basic studies	Dentistry degree (Bologna Plan)	76	24.9
	Dentistry degree (Old Plan)	171	56.1
	Stomatology	52	17.0
	Maxillofacial surgeon	6	2.0
Dental implant education	Master’s Degree students	16	5.2
	Postgraduate certificates	31	10.2
	Master’s Degree	174	57.0
	University Specialist Degree	84	27.5
Experience with dental implants (in years)	<5	57	18.7
	5–15	85	27.9
	15–20	52	17.0
	>20	111	36.4
Main number of dental implants placed per year	≤50	53	17.4
	51–100	65	21.3
	101–200	80	26.2
	>200	107	35.1
Exclusive clinical practice in dental implant treatments	Yes	43	14.1
	No	262	85.9
Job environment	Rural	35	11.5
	Urban	270	88.5
Where he/she performs his/her activity	Other dental practices	89	29.2
	Own dental practice	134	43.9
	Both	82	26.9
Works in several dental practices	Yes	196	64.3
	No	109	35.7
Weekly working hours	<16 h ^3^	4	1.3
	16–24 h	22	7.2
	25–32 h	54	17.7
	33–40 h	112	36.7
	>40 h	113	37.0

^1^ Participants; ^2^ percentage; ^3^ hours.

**Table 3:** Degrees of the dimensions examined in the MBI and the global prevalence of persons with BS.

Dimension	Grade	N ^1^	% ^2^	Mean ± SD ^3^
EE ^4^	Low	173	56.7	20.3 ± 13.8
	Average	45	14.8	
	High	87	28.5	
DE ^5^	Low	210	68.9	5.1 ± 5.9
	Average	50	16.4	
	High	45	14.8	
PA ^6^	Low	128	42.0	32.5 ± 14.5
	Average	38	12.5	
	High	139	45.6	
BS ^7^	Yes	13	4.3	
	No	292	95.7	
Signs of BS	No signs	106	34.8	
	One dimension	151	49.5	
	Two dimensions	35	11.5	

^1^ Participants; ^2^ percentage; ^3^ standard deviation; ^4^ emotional exhaustion; ^5^ depersonalization; ^6^ personal accomplishments; ^7^ burnout syndrome.

**Table 4:** Mean values of the questions asked in each dimension of the MBI (mean of the scores): 0 = never to 6 = every day).

Variable	Specification	Mean ± SD ^1^
EE ^2^	I feel emotionally drained by my job.	2.55 ± 1.72
	I feel tired at the end of the workday.	3.18 ± 1.79
	When I wake up in the morning and face another workday, I feel exhausted.	2.13 ± 1.77
	I consider that working all day with patients is a great effort and it tires me out.	2.68 ± 1.83
	I feel like my job is wearing me out. I feel burned out by my job.	2.31 ± 1.87
	I feel frustrated at my job.	1.60 ± 1.77
	I think I work too much.	2.60 ± 1.96
	Working directly with patients stresses me out.	2.16 ± 1.85
	I am exhausted at work, at the limit of my capabilities.	1.12 ± 1.56
DE ^3^	I believe I am treating certain patients as if they were impersonal objects.	0.69 ± 1.27
	I have become more insensitive to people since I started practicing as a dentist/maxillofacial surgeon.	1.04 ± 1.52
	I believe this job is hardening me emotionally.	1.29 ± 1.62
	I am not concerned about what happens to some of my patients.	0.55 ± 1.40
	I think the patients blame me for some of their problems.	1.52 ± 1.63
PA ^4^	I can easily comprehend how my patients feel.	4.28 ± 1.93
	I believe I handle my patients’ problems very effectively.	4.20 ± 1.86
	I feel that my work positively influences the lives of my patients.	4.17 ± 1.88
	My work makes me feel energised.	3.50 ± 1.93
	I am confident that I can easily create a pleasant atmosphere for my patients.	4.08 ± 1.90
	I feel motivated after working with my patients.	3.59 ± 1.98
	I believe I accomplish a lot of worthwhile things at work.	3.61 ± 1.97
	In my job, I deal with emotional issues in a very calm manner.	3.54 ± 1.99

^1^ Standard deviation; ^2^ emotional exhaustion; ^3^ depersonalization; ^4^ personal accomplishments.

## Data Availability

The data that supports the findings of this study are available on from the corresponding author upon reasonable request.

## Supplementary Materials

The following supporting information can be downloaded at: https://www.mdpi.com/article/10.3390/healthcare13141724/s1, Table S1: Questionnaire used in the present study.

## Abbreviations

The following abbreviations are used in this manuscript:

BS	Burnout syndrome
EE	Emotional exhaustion
DE	Depersonalization
PA	Personal accomplishment
ICD-11	11th Revision of the International Classification of Diseases
STROBE	Strengthening the Reporting of Observational Studies in Epidemiology
SEI	Spanish Society of Implants
MBI–HSS	Maslach Burnout Inventory—Human Services Survey
MS	Maximum score
h	Hour(s)
SD	Standard deviation
OR	Odds ratio
UWES	Utrecht Work Engagement Scale
r	Pearson correlation coefficient
vs.	Versus
