# Supplementary material for: Burnout Syndrome Among Spanish Professionals Dedicated to Implant Dentistry: An Observational Study
Source: Healthcare (Basel). 2025 Jul 17;13(14):1724. doi: 10.3390/healthcare13141724 (PMC12294223; doi:10.3390/healthcare13141724)
Supplement: Supplementary file 1 [file healthcare-13-01724-s001.zip › healthcare-3730783-supplementary.pdf]

Table S1. Questionnaire used in the present study

BLOCK I
General information

1) **Gender:**
   a) Male.
   b) Female.
2) **Age (years):**
   a) ≤ 30
   b) 31 – 40
   c) 41 – 50
   d) 51 – 60
   e) > 60
3) **Level of university education**
   a) Dentistry degree (Bologna Plan)
   b) Dentistry degree (Old Plan)
   c) Stomatology
   d) Maxillofacial surgeon
4) **Postgraduate level of education attained.**
   a) Non-accredited training courses (clinical stays, commercial firm courses, etc)
   b) University postgraduate degrees related to Oral Implantology
   c) Student of a university master's program related to Oral Implantology.
   d) Having completed a university master's degree related to Oral Implantology.
5) **Experience placing implants (years**).
   a) ≤ 5
   b) 5 – 15
   c) 15 – 20
   d) > 20
6) **Average number of implants placed per year**
   a) ≤ 50
   b) 51 – 100
   c) 101 – 200
   d) > 200
7) **Exclusive scope of practice in implant placement procedures**
   a) Yes
   b) No
8) **Workplace**
   a) Rural
   b) Urban
9) **How the work is performed**
   a) I work for other dental practices
   b) I own my dental practice
   c) Both
10) **Work in several workplaces**
   a) Yes
   b) No
11) **Number of working hours (h) per week**
   a) < 16 h
   b) 16 a 24 h
   c) 25 – 32 h
   d) 33 – 40 h
   e) > 40 h

BLOCK II
Burnout Questionnaire

EE – Evaluation of emotional exhaustion.

Affirmations	Degree of agreement (Assigned value)
1) I feel emotionally drained by my job.	Never (0)<br>Few times a year (1)<br>Once or twice a month (2)<br>Few times a month (3)<br>Once a week (4)<br>Few times a week (5)<br>Every day (6)
2) I feel tired at the end of the workday.	
3) When I wake up in the morning and face another workday, I feel exhausted.	
4) I consider that working all day with patients is a great effort and it tires me out.	
5) I feel like my job is wearing me out. I feel burned out by my job.	
6) I feel frustrated at my job.	
7) I think I work too much.	
8) Working directly with patients stresses me out.	
9) I'm exhausted at work, at the limit of my capabilities.	

PA – Evaluation of depersonalisation

Affirmations	Degree of agreement (Assigned value)
1) I believe I am treating certain patients as if they were impersonal objects.	Never (0)<br>Few times a year (1)<br>Once or twice a month (2)<br>Few times a month (3)<br>Once a week (4)<br>Few times a week (5)<br>Every day (6)
2) I have become more insensitive to people since I started practicing as a dentist.	
3) I believe this job is hardening me emotionally.	
4) I'm not concerned about what happens to some of my patients	
5) I think the patients blame me for some of their problems.	

PA – Evaluation of personal fulfilment.

Affirmations	Degree of agreement (Assigned value)
1) I can easily comprehend how my patients feel.	Never (0)<br>Few times a year (1)<br>Once or twice a month (2)<br>Few times a month (3)<br>Once a week (4)<br>Few times a week (5)<br>Every day (6)
2) I believe I handle my patients' problems very effectively.	
3) I feel that my work positively influences the lives of my patients.	
4) My work makes me feel energised.	
5) I'm confident that I can easily create a pleasant atmosphere for my patients.	
6) I feel motivated after working with my patients.	
7) I believe I accomplish a lot of worthwhile things at work.	
8) In my job, I deal with emotional issues in a very calm manner	
